# Poly[dimethano­lbis[μ-5-(3-pyrid­yl)tetra­zolato-κ^2^ *N* ^2^:*N* ^5^]copper(II)]

**DOI:** 10.1107/S1600536810013553

**Published:** 2010-04-21

**Authors:** Xiao-Hong Wei

**Affiliations:** a College of Mechanical & Material Engineering, China Three Gorges University, Yichang 443002, People’s Republic of China

## Abstract

In the crystal structure of the title complex, [Cu(C_6_H_4_N_5_)_2_(CH_3_OH)_2_]_*n*_, the Cu^II^ cation lies on an inversion center and is coordinated by four 5-(3-pyrid­yl)tetra­zolate anions and two methanol mol­ecules in an elongated distorted CuN_4_O_2_ octa­hedral geometry. Each 5-(3-pyrid­yl)tetra­zolate anion bridges two Cu^II^ cations, forming a two-dimensional polymeric complex with (4,4) network topology. In the crystal structure, the two-dimensional layers are connected by inter­molecular O—H⋯N hydrogen bonding, forming a three-dimensional supra­molecular architecture.

## Related literature

For background to 5-(3-pyrid­yl)tetra­zolate complexes, see: Fu *et al.* (2008 ▶); Wang *et al.* (2005 ▶). For the structure of a related polymeric metal complex with a 5-(3-pyrid­yl)tetra­zolate bridging ligand, see: Zhang *et al.* (2006 ▶).
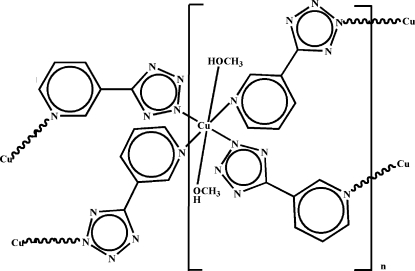


## Experimental

### 

#### Crystal data

[Cu(C_6_H_4_N_5_)_2_(CH_4_O)_2_]
*M* *_r_* = 419.91
Orthorhombic, 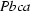

*a* = 13.553 (3) Å
*b* = 9.1756 (18) Å
*c* = 14.264 (3) Å
*V* = 1773.8 (6) Å^3^
*Z* = 4
Mo *K*α radiation
μ = 1.27 mm^−1^
*T* = 298 K
0.35 × 0.23 × 0.20 mm

#### Data collection

Bruker SMART CCD diffractometer
Absorption correction: multi-scan (*SADABS*; Sheldrick, 1996 ▶) *T*_min_ = 0.712, *T*_max_ = 0.776
10117 measured reflections
2142 independent reflections
1609 reflections with *I* > 2σ(*I*)
*R*_int_ = 0.034

#### Refinement

*R*[*F* ^2^ > 2σ(*F* ^2^)] = 0.031
*wR*(*F* ^2^) = 0.102
*S* = 1.00
2142 reflections
126 parameters
H-atom parameters constrained
Δρ_max_ = 0.34 e Å^−3^
Δρ_min_ = −0.44 e Å^−3^

### 

Data collection: *SMART* (Bruker, 2007 ▶); cell refinement: *SAINT* (Bruker, 2007 ▶); data reduction: *SAINT*; program(s) used to solve structure: *SHELXTL* (Sheldrick, 2008 ▶); program(s) used to refine structure: *SHELXTL*; molecular graphics: *SHELXTL*; software used to prepare material for publication: *SHELXTL*.

## Supplementary Material

Crystal structure: contains datablocks I, global. DOI: 10.1107/S1600536810013553/xu2736sup1.cif

Structure factors: contains datablocks I. DOI: 10.1107/S1600536810013553/xu2736Isup2.hkl

Additional supplementary materials:  crystallographic information; 3D view; checkCIF report

## Figures and Tables

**Table 1:** Selected bond lengths (Å)

Cu1—N1^i^	2.0549 (16)
Cu1—N3	2.0167 (15)
Cu1—O1	2.4999 (15)

Symmetry code: (i) 
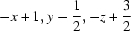
.

**Table 2:** Hydrogen-bond geometry (Å, °)

*D*—H⋯*A*	*D*—H	H⋯*A*	*D*⋯*A*	*D*—H⋯*A*
O1—H1*A*⋯N5^ii^	0.82	1.97	2.776 (2)	166

Symmetry code: (ii) 
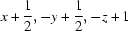
.

**Fig. 1.**
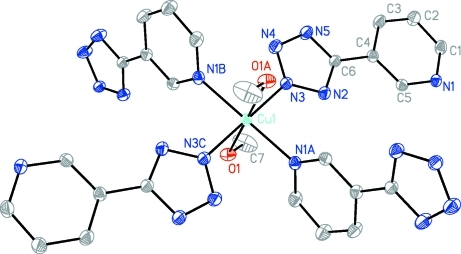
Part of the polymeric structure of the title compound. Displacement ellipsoids are drawn at the 30% probability level. [Symmetry code: (A) -x+1,y-1/2,-z+3/2; (B) x,-y+1/2,z-1/2; (C) -x+1,-y,-z+1.]

**Fig. 2.**
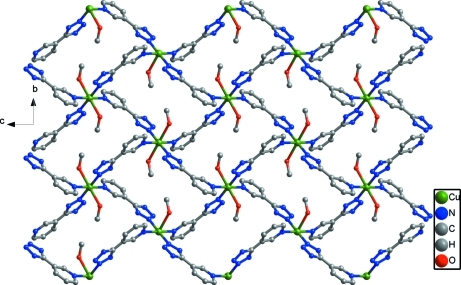
The 2D sheet of the title compound, viewed along the a axis.

## supplementary crystallographic information

### Comment

In recent years, there has been great interest in the study of metal-organic coordination polymers with network structures due to their possible chemical and physicalproperties. Tetrazole compounds are a class of excellent ligands for the construction of novel metal-organic frameworks, dueing to its various coordination modes (Wang *et al.*, 2005; Fu *et al.*, 2008; Zhang *et al.*,  2006)). We report here the crystal structure of the title compound.

The crystallographically asymmetric unit contains a half Cu^II^ ion, one 5-(3-pyridyl)tetrazolate (3-ptz) ligand and one methanol molecule. Each ligand adopts a bidentate bridging spacer to link two Cu centers. Upon the same bridging fashion, all Cu^II^ atoms are linked by ligands into a infinite 2D grid network with Cu···Cu separation of 8.480 (2) Å. In additon, the pyridyl and tetrazolate rings are almost coplanar, with a dihedral angle of 2.535 (7)°. The bond distances between Cu and N atoms are in the range of 2.017 (2)-2.055 (2) Å. There is intermolecular O—H···N hydrogen bond inginteractions involving the hydroxyl of methanol and nitrogen of 3-ptz ligand, which links the 2D layers into a 3D supramolecular architecture.

### Experimental

A mixture of 3-(2*H*-tetrazol-5-yl)pyridine(0.2 mmol, 0.0294 g),Cu(CH_3_COO)_2_.H_2_O (0.4 mmol, 0.0799 g), methanol (5 ml) and distilled water (10 ml) were sealed in a 25 ml Teflon-lined stainless steel reactor and heated at 423 K for three days, and then cooled slowly to 298 K at which time blue crystals were obtained.

### Refinement

H atoms were positioned geometrically (C—H = 0.93 and 0.96 Å, O—H = 0.82 Å), and allowed to ride on their parent atoms, with *U*_iso_(H) = 1.5*U*_eq_(C) for methyl or 1.2*U*_eq_(C,O) for the others.

### Crystal data

**Table:**

[Cu(C_6_H_4_N_5_)_2_(CH_4_O)_2_]	*F*(000) = 860
*M**_r_* = 419.91	*D*_x_ = 1.572 Mg m^−^^3^
Orthorhombic, *P**b**c**a*	Mo *K*α radiation, λ = 0.71073 Å
Hall symbol: -P 2ac 2ab	Cell parameters from 3436 reflections
*a* = 13.553 (3) Å	θ = 3.0–28.4°
*b* = 9.1756 (18) Å	µ = 1.27 mm^−^^1^
*c* = 14.264 (3) Å	*T* = 298 K
*V* = 1773.8 (6) Å^3^	Prism, blue
*Z* = 4	0.35 × 0.23 × 0.20 mm

### Data collection

**Table:**

Bruker SMART CCD diffractometer	2142 independent reflections
Radiation source: fine-focus sealed tube	1609 reflections with *I* > 2σ(*I*)
graphite	*R*_int_ = 0.034
φ and ω scans	θ_max_ = 28.4°, θ_min_ = 3.0°
Absorption correction: multi-scan (*SADABS*; Sheldrick, 1996)	*h* = −17→15
*T*_min_ = 0.712, *T*_max_ = 0.776	*k* = −11→10
10117 measured reflections	*l* = −10→19

### Refinement

**Table:**

Refinement on *F*^2^	Primary atom site location: structure-invariant direct methods
Least-squares matrix: full	Secondary atom site location: difference Fourier map
*R*[*F*^2^ > 2σ(*F*^2^)] = 0.031	Hydrogen site location: inferred from neighbouring sites
*wR*(*F*^2^) = 0.102	H-atom parameters constrained
*S* = 1.00	*w* = 1/[σ^2^(*F*_o_^2^) + (0.063*P*)^2^ + 0.5194*P*] where *P* = (*F*_o_^2^ + 2*F*_c_^2^)/3
2142 reflections	(Δ/σ)_max_ = 0.006
126 parameters	Δρ_max_ = 0.34 e Å^−^^3^
0 restraints	Δρ_min_ = −0.44 e Å^−^^3^

### Special details

**Table:**

Geometry. All e.s.d.'s (except the e.s.d. in the dihedral angle between two l.s. planes) are estimated using the full covariance matrix. The cell e.s.d.'s are taken into account individually in the estimation of e.s.d.'s in distances, angles and torsion angles; correlations between e.s.d.'s in cell parameters are only used when they are defined by crystal symmetry. An approximate (isotropic) treatment of cell e.s.d.'s is used for estimating e.s.d.'s involving l.s. planes.
Refinement. Refinement of *F*^2^ against ALL reflections. The weighted *R*-factor wR and goodness of fit *S* are based on *F*^2^, conventional *R*-factors *R* are based on *F*, with *F* set to zero for negative *F*^2^. The threshold expression of *F*^2^ > σ(*F*^2^) is used only for calculating *R*-factors(gt) etc. and is not relevant to the choice of reflections for refinement. *R*-factors based on *F*^2^ are statistically about twice as large as those based on *F*, and *R*- factors based on ALL data will be even larger.

### Fractional atomic coordinates and isotropic or equivalent isotropic displacement parameters (Å^2^)

**Table:**

	*x*	*y*	*z*	*U*_iso_*/*U*_eq_	
Cu1	0.5000	0.0000	0.5000	0.02552 (13)	
N1	0.40143 (12)	0.49681 (14)	0.89055 (11)	0.0268 (3)	
N2	0.43581 (11)	0.21166 (17)	0.65120 (11)	0.0322 (4)	
C4	0.34156 (12)	0.39225 (18)	0.74718 (13)	0.0271 (4)	
C6	0.35651 (13)	0.29258 (19)	0.66749 (12)	0.0273 (4)	
N3	0.41335 (10)	0.13933 (17)	0.57196 (11)	0.0289 (3)	
N5	0.28715 (12)	0.2724 (2)	0.60197 (12)	0.0400 (4)	
C5	0.41360 (13)	0.41125 (19)	0.81498 (12)	0.0278 (4)	
H5	0.4733	0.3624	0.8078	0.033*	
C1	0.31558 (14)	0.5690 (2)	0.89861 (14)	0.0356 (4)	
H1	0.3065	0.6299	0.9500	0.043*	
C2	0.24063 (16)	0.5566 (3)	0.83412 (14)	0.0417 (5)	
H2	0.1822	0.6081	0.8421	0.050*	
N4	0.32433 (12)	0.17505 (19)	0.54228 (12)	0.0397 (4)	
C3	0.25302 (14)	0.4669 (3)	0.75747 (13)	0.0381 (5)	
H3	0.2030	0.4566	0.7134	0.046*	
O1	0.58452 (9)	0.21181 (15)	0.42449 (11)	0.0392 (3)	
H1A	0.6426	0.2084	0.4085	0.047*	
C7	0.5580 (2)	0.3571 (3)	0.4431 (3)	0.0756 (10)	
H7A	0.4874	0.3658	0.4430	0.113*	
H7B	0.5853	0.4194	0.3956	0.113*	
H7C	0.5832	0.3853	0.5033	0.113*	

### Atomic displacement parameters (Å^2^)

**Table:**

	*U*^11^	*U*^22^	*U*^33^	*U*^12^	*U*^13^	*U*^23^
Cu1	0.0267 (2)	0.0285 (2)	0.02140 (19)	0.00631 (11)	−0.00140 (11)	−0.00145 (11)
N1	0.0310 (8)	0.0256 (8)	0.0238 (7)	−0.0035 (6)	−0.0006 (6)	0.0000 (6)
N2	0.0308 (7)	0.0373 (9)	0.0286 (8)	0.0049 (7)	−0.0035 (6)	−0.0083 (7)
C4	0.0300 (8)	0.0257 (8)	0.0257 (8)	0.0025 (7)	0.0001 (7)	−0.0014 (7)
C6	0.0268 (8)	0.0289 (9)	0.0261 (8)	0.0016 (7)	−0.0026 (7)	−0.0013 (7)
N3	0.0270 (7)	0.0322 (8)	0.0274 (7)	0.0030 (6)	−0.0025 (6)	−0.0029 (6)
N5	0.0332 (8)	0.0492 (10)	0.0376 (9)	0.0138 (7)	−0.0090 (7)	−0.0173 (8)
C5	0.0268 (8)	0.0290 (9)	0.0275 (9)	0.0017 (7)	0.0011 (7)	−0.0004 (7)
C1	0.0423 (10)	0.0348 (10)	0.0296 (10)	0.0069 (9)	−0.0016 (8)	−0.0063 (8)
C2	0.0411 (11)	0.0452 (12)	0.0387 (11)	0.0195 (10)	−0.0063 (9)	−0.0102 (9)
N4	0.0328 (8)	0.0480 (10)	0.0382 (9)	0.0121 (7)	−0.0076 (7)	−0.0149 (8)
C3	0.0361 (11)	0.0442 (11)	0.0339 (11)	0.0122 (8)	−0.0109 (9)	−0.0082 (8)
O1	0.0296 (7)	0.0388 (8)	0.0492 (9)	−0.0034 (6)	−0.0003 (6)	0.0014 (6)
C7	0.0670 (17)	0.0381 (14)	0.122 (3)	0.0021 (13)	0.0290 (18)	0.0100 (15)

### Geometric parameters (Å, °)

**Table:**

Cu1—N1^i^	2.0549 (16)	C6—N5	1.338 (2)
Cu1—N1^ii^	2.0549 (16)	N3—N4	1.320 (2)
Cu1—N3	2.0167 (15)	N5—N4	1.333 (2)
Cu1—N3^iii^	2.0167 (15)	C5—H5	0.9300
Cu1—O1	2.4999 (15)	C1—C2	1.375 (3)
Cu1—O1^iii^	2.4999 (15)	C1—H1	0.9300
N1—C5	1.344 (2)	C2—C3	1.378 (3)
N1—C1	1.344 (2)	C2—H2	0.9300
N1—Cu1^iv^	2.0549 (16)	C3—H3	0.9300
N2—C6	1.327 (2)	O1—C7	1.406 (3)
N2—N3	1.346 (2)	O1—H1A	0.8200
C4—C5	1.385 (2)	C7—H7A	0.9600
C4—C3	1.390 (2)	C7—H7B	0.9600
C4—C6	1.473 (2)	C7—H7C	0.9600
			
N3—Cu1—N3^iii^	180.00 (7)	N1—C5—C4	123.17 (16)
N3—Cu1—N1^i^	90.06 (6)	N1—C5—H5	118.4
N3^iii^—Cu1—N1^i^	89.94 (6)	C4—C5—H5	118.4
N3—Cu1—N1^ii^	89.94 (6)	N1—C1—C2	122.80 (18)
N3^iii^—Cu1—N1^ii^	90.06 (6)	N1—C1—H1	118.6
N1^i^—Cu1—N1^ii^	180.0	C2—C1—H1	118.6
C5—N1—C1	117.57 (16)	C3—C2—C1	119.33 (18)
C5—N1—Cu1^iv^	122.55 (12)	C3—C2—H2	120.3
C1—N1—Cu1^iv^	119.40 (13)	C1—C2—H2	120.3
C6—N2—N3	103.87 (14)	N3—N4—N5	107.89 (15)
C5—C4—C3	118.22 (16)	C2—C3—C4	118.89 (17)
C5—C4—C6	121.33 (16)	C2—C3—H3	120.6
C3—C4—C6	120.42 (16)	C4—C3—H3	120.6
N2—C6—N5	111.67 (15)	C7—O1—H1A	109.5
N2—C6—C4	126.44 (16)	O1—C7—H7A	109.5
N5—C6—C4	121.88 (16)	O1—C7—H7B	109.5
N4—N3—N2	110.72 (14)	H7A—C7—H7B	109.5
N4—N3—Cu1	121.77 (12)	O1—C7—H7C	109.5
N2—N3—Cu1	127.47 (11)	H7A—C7—H7C	109.5
N4—N5—C6	105.85 (15)	H7B—C7—H7C	109.5
			
N3—N2—C6—N5	0.1 (2)	C4—C6—N5—N4	−179.06 (17)
N3—N2—C6—C4	179.14 (17)	C1—N1—C5—C4	−1.4 (3)
C5—C4—C6—N2	0.5 (3)	Cu1^iv^—N1—C5—C4	170.55 (13)
C3—C4—C6—N2	−177.71 (19)	C3—C4—C5—N1	0.8 (3)
C5—C4—C6—N5	179.43 (18)	C6—C4—C5—N1	−177.41 (16)
C3—C4—C6—N5	1.2 (3)	C5—N1—C1—C2	1.1 (3)
C6—N2—N3—N4	−0.2 (2)	Cu1^iv^—N1—C1—C2	−171.19 (17)
C6—N2—N3—Cu1	177.41 (12)	N1—C1—C2—C3	−0.1 (4)
N3^iii^—Cu1—N3—N4	12 (76)	N2—N3—N4—N5	0.2 (2)
N1^i^—Cu1—N3—N4	−171.52 (15)	Cu1—N3—N4—N5	−177.56 (13)
N1^ii^—Cu1—N3—N4	8.48 (15)	C6—N5—N4—N3	−0.1 (2)
N3^iii^—Cu1—N3—N2	−165 (76)	C1—C2—C3—C4	−0.5 (4)
N1^i^—Cu1—N3—N2	11.10 (15)	C5—C4—C3—C2	0.2 (3)
N1^ii^—Cu1—N3—N2	−168.90 (15)	C6—C4—C3—C2	178.4 (2)
N2—C6—N5—N4	0.0 (2)		

Symmetry codes: (i) −*x*+1, *y*−1/2, −*z*+3/2; (ii) *x*, −*y*+1/2, *z*−1/2; (iii) −*x*+1, −*y*, −*z*+1; (iv) −*x*+1, *y*+1/2, −*z*+3/2.

### Hydrogen-bond geometry (Å, °)

**Table:**

*D*—H···*A*	*D*—H	H···*A*	*D*···*A*	*D*—H···*A*
O1—H1A···N5^v^	0.82	1.97	2.776 (2)	166

Symmetry codes: (v) *x*+1/2, −*y*+1/2, −*z*+1.
